# A novel hybrid algorithm based on Harris Hawks for tumor feature gene selection

**DOI:** 10.7717/peerj-cs.1229

**Published:** 2023-02-13

**Authors:** Junjian Liu, Huicong Feng, Yifan Tang, Lupeng Zhang, Chiwen Qu, Xiaomin Zeng, Xiaoning Peng

**Affiliations:** 1 Department of Statistics, Hunan Normal University College of Mathematics and Statistics, Changsha, Hunan, China; 2 Department of Pathology and Pathophysiology, Hunan Normal University School of Medicine, Changsha, Hunan, China; 3 Department of Biochemistry and Molecular Biology, Jishou University School of Medicine, Jishou, Hunan, China; 4 Department of Epidemiology and Health Statistics, Xiangya Public Health School, Central South University, Changsha, Hunan, China

**Keywords:** Gene selection, Microarray gene expression, Harris Hawks algorithm, Extremely randomized tree

## Abstract

**Background:**

Gene expression data are often used to classify cancer genes. In such high-dimensional datasets, however, only a few feature genes are closely related to tumors. Therefore, it is important to accurately select a subset of feature genes with high contributions to cancer classification.

**Methods:**

In this article, a new three-stage hybrid gene selection method is proposed that combines a variance filter, extremely randomized tree and Harris Hawks (VEH). In the first stage, we evaluated each gene in the dataset through the variance filter and selected the feature genes that meet the variance threshold. In the second stage, we use extremely randomized tree to further eliminate irrelevant genes. Finally, we used the Harris Hawks algorithm to select the gene subset from the previous two stages to obtain the optimal feature gene subset.

**Results:**

We evaluated the proposed method using three different classifiers on eight published microarray gene expression datasets. The results showed a 100% classification accuracy for VEH in gastric cancer, acute lymphoblastic leukemia and ovarian cancer, and an average classification accuracy of 95.33% across a variety of other cancers. Compared with other advanced feature selection algorithms, VEH has obvious advantages when measured by many evaluation criteria.

## Introduction

In data analysis, data dimension may be much more than the number of samples (Diao & Vidyashankar, 2013). The generally-used methods often perform poorly on such data, because they can-not avoid the dimensionality curse (Myakalwar et al., 2015). Therefore, it is necessary to datasets with feature that ensure the accuracy of subsequent analysis (Douglas & Shapiro, 2021). Microarray technology can simultaneously measure a large number of cancer related gene expression data (Sandra, Shukla & Kolthur-Seetharam, 2020; Su et al., 2017), and the efficient selection of disease feature genes from microarray data can improve the accuracy of disease classification and help to improve the treatment of cancer (An, Wang & Wei, 2018). Because the number of gene expressions is much larger than the number of cancer samples, and only a few feature genes in the gene expression data are closely related to cancers, selecting highly discriminative feature genes for cancers is a challenging task, and the existing methods are not effective.

Genes are closely related to tumors. Gene activation and mutation are one of the causes of tumor occurrence. Feature selection of high-dimensional data is divided into four standard methods: filter, embedded, wrapper, and hybrid (Dashtban & Balafar, 2017; Pashaei & Pashaei, 2021; Pfeifer et al., 2020; Tang et al., 2021; Yu & Ni, 2014). The filter method is a preprocessing method used for high-dimensional data. It evaluates each gene in the tumor according to specific rules and removes genes unrelated to the follow-up learning process. However, the filter method cannot analyze the mutual information between features, and the selected feature subset may not be optimal (Wang, Wang & Chang, 2016). The wrapper method uses the classification model including the heuristic algorithm and selects the optimal feature subset according to the classification performance (Sahebi et al., 2020). In the feature selection of high-dimensional medical data, the wrapper method is usually more effective than the filter method (Fu et al., 2020). The hybrid method is the combined application of the filter, embedded, and wrapper methods, as well as the improvement and expansion based on these three methods (Castellanos-Garzón et al., 2018). For example, Qu et al. (2021) proposed the Harris Hawks optimizer with variable neighborhood to screen feature genes, by combining the wrapper and embedded methods. Zhang et al. (2021) proposed a hybrid method that combined a Fisher score and gradient enhanced decision tree, and selected the best feature gene set with robustness across 11 high-dimensional gene expression datasets. Chuang et al. (2009), Deng et al. (2022), and Mandal et al. (2021) also adopted the hybrid method by combining the filter and wrapper methods, and achieved good results in multiple open cancer datasets. This article presents a three-stage feature selection hybrid method VEH, that combines the filter and wrapper methods. Through the analysis and comparison of the experimental results, we confirmed that the VEH method has obvious advantages in the selection performance of feature genes, number of selected genes and calculation time.

The chapters of this article are organized as follows: first, we summarize the research work and corresponding algorithm principles related to variance filter, extremely randomized tree, Harris Hawks algorithm and introduce the hybrid algorithm VEH in detail. In the result, we compared the VEH method with 13 related feature selection algorithms, based on eight published cancer gene expression datasets. Finally, we summarize the experimental results.

## Materials & Methods

### Variance filter

Variance is important when measuring the degree of data dispersion. Hackstadt & Hess (2009) studied in detail the effect of using a variance filter on microarray data analysis. In this article, we set the variance threshold to 0.05 to filter out all feature genes whose variance was less than the threshold.

### Extremely randomized tree

The extremely randomized tree is a machine learning algorithm constructed from multiple decision trees (Liang et al., 2021). Extremely randomized trees have the advantages of high computing efficiency and are suitable for processing high dimensional data. Extremely randomized trees constructs decision trees by randomly selecting attributes and splitting nodes.

### Harris Hawks algorithm

**Figure 1:**
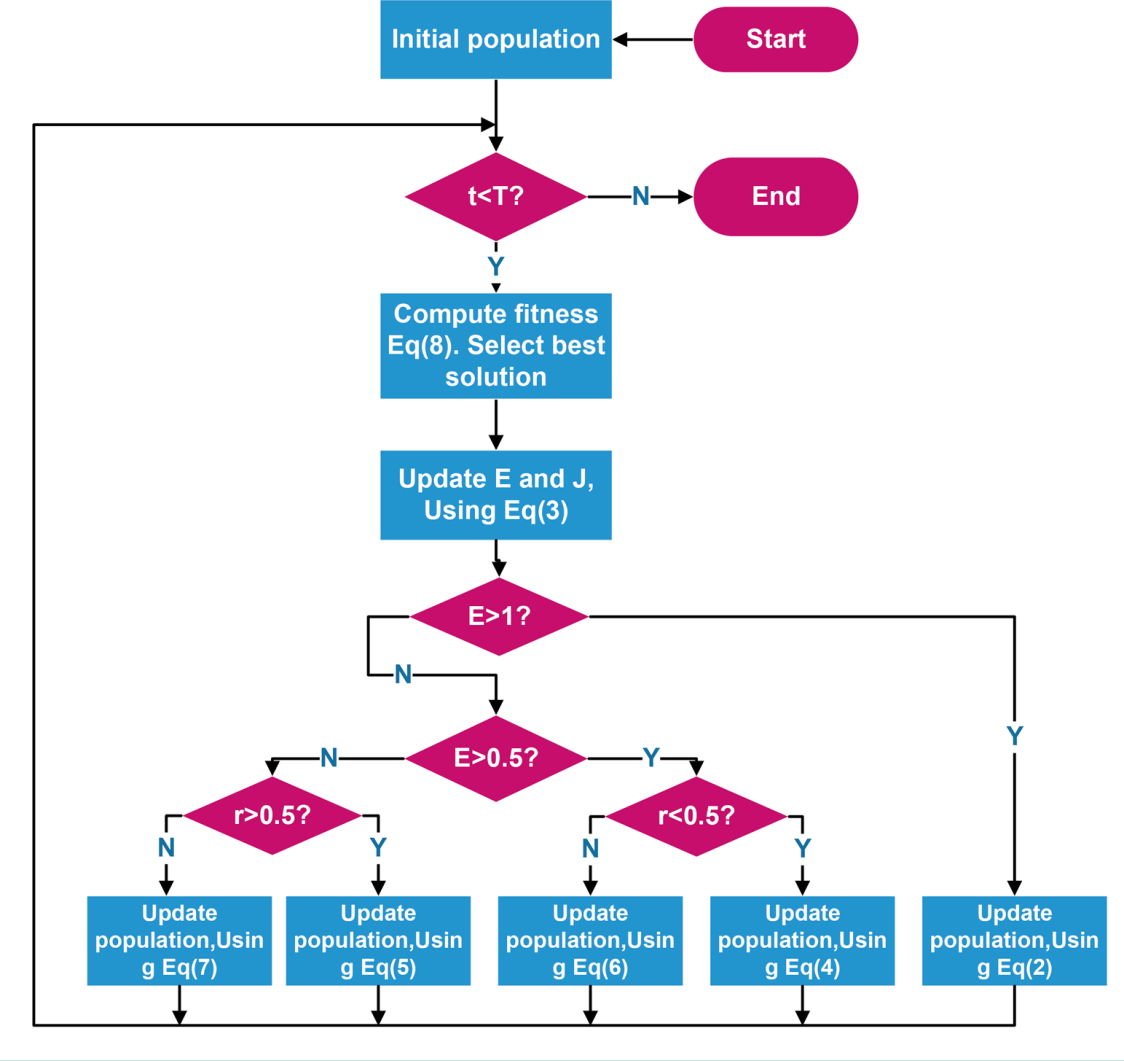
HHO workflow.

Heidari et al. (2019) proposed the Harris Hawks algorithm (HHO) according to the hunting law of Harris hawks in nature. The Harris Hawks algorithm is a new type of swarm intelligence optimization algorithm, that has strong search ability and high accuracy. In the algorithm, prey *rabbit* represents the fitness optimal solution in the current iteration. The whole algorithm is divided into two stages: exploratory and development. The exploratory stage starts by initializing a value to detect the habitat position and then observing the prey. During the development stage, the Harris Hawks carry out four attack modes based on the energy of their prey and the possibility of escape. Figure 1 shows the HHO workflow.

During the exploratory stage, the escape energy factor is *E*. When $ \left\vert E \right\vert \geq 1$, Harris Hawks randomly searches $ \left[ lb,ub \right] $ and uses two strategies with the same probability to search for prey globally. The location update formula is shown in Eq. (1):

(1) \begin{eqnarray*}X(t+1)= \left\{ \begin{array}{@{}cc@{}} \displaystyle {X}_{rand}(t)-{r}_{1} \left\vert {X}_{rand}(t)-2{r}_{2}X(t) \right\vert &\displaystyle q\geq 0.5\\ \displaystyle ({X}_{rabbit}(t)-{X}_{m}(t))-{r}_{3}(lb+{r}_{4}(ub-lb))&\displaystyle q\lt 0.5 \end{array} \right. \end{eqnarray*}

where, X_rand_ is the randomly selected Harris Hawks position in the population; *X*(*t*) is the individual position in the iteration; *X*_*rabbit*_ is the prey position in the current iteration; *X*_*m*_ is the average position information of the population; and r_1_, r_2_, r_3_, r_4_ is a random number with (0, 1) distribution. *q* is the conversion factor controlling the two strategies.

HHO controls the exploratory and development stages through the escape energy factor, as shown in Eq. (2):

(2) \begin{eqnarray*}\mathrm{E}=2{E}_{0} \left( 1- \frac{t}{T} \right) \end{eqnarray*}

where *T* is the total number of iterations and *E*_0_ is the random number of initial energy values (−1, 1). When $ \left\vert \mathrm{E} \right\vert \geq 1$, a global search is performed; otherwise, the development stage begins.

During the development stage, when $ \left\vert \mathrm{E} \right\vert \lt 1$, the Harris Hawks raid and catch prey, and the prey avoids predation. HHO is based on random numbers *r* ∈ (0, 1), *E* and the appropriate one is selected from the following four attack strategies to complete the location update where |*E*| is the deciding factor of the strategy. When |E| ≥ 0.5, Harris Hawks selects the soft besiege strategy; otherwise, it selects the hard besiege strategy. *r* is the probability of prey being captured.

1. When *r* ≥ 0.5, Harris Hawks can capture prey; otherwise, the hunt fails. When *r* ≥ 0.5 and $ \left\vert E \right\vert \geq 0.5$, the prey jumps with sufficient energy to avoid predation, and Harris Hawks uses prey energy to complete its predation using a soft besiege strategy, as shown in Formula (3):
  (3) \begin{eqnarray*} \left\{ \begin{array}{@{}c@{}} \displaystyle {X}_{(t+1)}=D(t)-E \left\vert J{X}_{\mathit{rabbit}}(t)-{X}_{ \left( t \right) } \right\vert \\ \displaystyle D(t)={X}_{\mathit{rabbit}}(t)-X(t)\\ \displaystyle J=2(1-{r}_{5}) \end{array} \right. \end{eqnarray*}
  where, *r*_5_ is a random number in (0, 1), *D*(*t*) is the distance between the prey and the current individual, and *J* is the movement distance of prey in jumping mode.
2. When *r* ≥ 0.5 and $ \left\vert E \right\vert \lt 0.5$, prey energy is insufficient, Harris Hawks carries out a hard besiege strategy and quickly preys, as shown in Formula (4):
  (4) \begin{eqnarray*}\mathrm{X}(\mathrm{t}+1)={X}_{\mathit{rabbit}}(t)-E \left\vert D(t) \right\vert \end{eqnarray*}
3. When *r* < 0.5, and |*E*| ≥ 0.5, the prey has enough energy to escape. At this time, Harris Hawks selects the soft besiege with progressive rapid dives strategy, as shown in Formula (5). This strategy has two hunting methods. When the first fails, the second is chosen.
  (5) \begin{eqnarray*}X(t+1)= \left\{ \begin{array}{@{}cc@{}} \displaystyle Y:{X}_{\mathit{rabbit}}(t)-E \left\vert J{X}_{\mathit{rabbit}}(t)-X(t) \right\vert &\displaystyle f(Y)\lt f(X(t))\\ \displaystyle Z:Y+S\times LF(D)&\displaystyle f(Z)\lt f(X(t)) \end{array} \right. \end{eqnarray*}
  where *S* is a random vector, *D* is the spatial dimension, *f* is the fitness function, and *LF* is the levy function, simulating the jumping behavior of prey.
4. When *r* < 0.5 and $ \left\vert E \right\vert \lt 0.5$, prey lacks energy but has a chance to escape. At this time, Harris Hawks chooses the hard besiege with progressive rapid dives strategy to narrow the distance from prey and form an encirclement circle, as shown in Formula (6):
  (6) \begin{eqnarray*}X(t+1)= \left\{ \begin{array}{@{}cc@{}} \displaystyle Y:{X}_{\mathit{rabbit}}(t)-E \left\vert J{X}_{\mathit{rabbit}}(t)-X(t) \right\vert &\displaystyle f(Y)\lt f(X(t))\\ \displaystyle Z:Y+S\times LF(D)&\displaystyle f(Z)\lt f(X(t)) \end{array} \right. \end{eqnarray*}

### Coding rules

When the HHO algorithm searches the optimal feature gene subset, it needs to encode the feature dimensions of all feasible solutions in HHO using binary string to solve the discrete space optimization problem. We used 1 and 0 to represent the retention and elimination of the gene respectively, set the value range of the feature as [0, 1], and updated the value of the binary coding position using the rounding method.

### Fitness function

The fitness function is used to evaluate the advantages and disadvantages of individuals and determine the optimization direction of the algorithm. We selected KNN as the fitness function of the classification problem, as shown in Formula (7):

(7) \begin{eqnarray*}\mathit{fitness}=\alpha (1-KN{N}_{acc})+(1-\alpha ) \frac{{f}_{num}}{{F}_{num}} \end{eqnarray*}

where, *KNN*_*acc*_ is the classification accuracy using the KNN classifier, *num*_*c*_ is the correct classification quantity, *num*_*e*_ is the number of wrong classifications, *f*_*num*_ and *F*_*num*_ are the feature subset and total feature number respectively, and *α* is an adjustment parameter (we set *α* = 0.99).

### VEH

**Figure 2:**
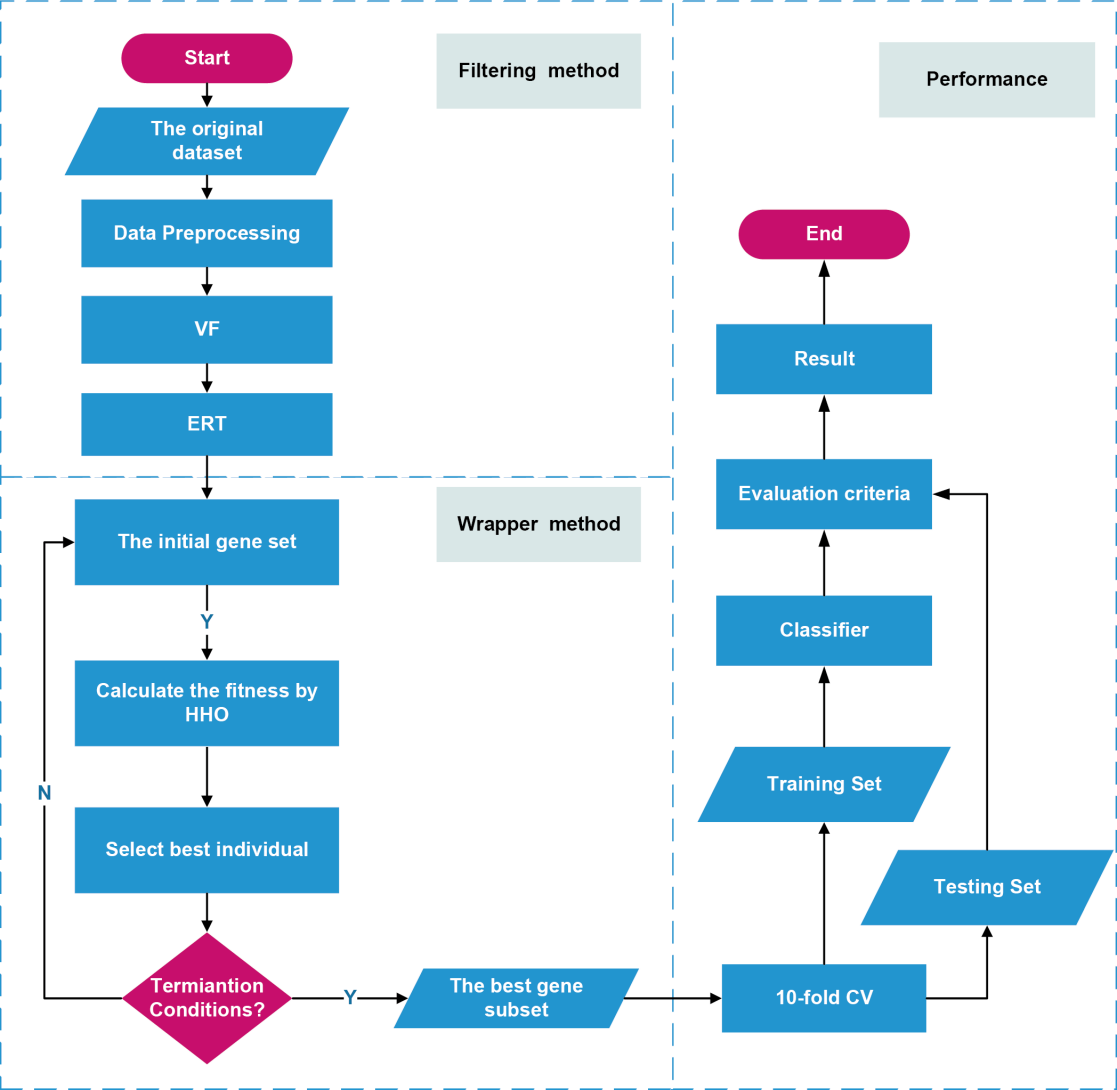
VEH workflow.

In this article, we propose a three-stage gene selection method, VEH, which combines a variance filter, extremely randomized tree, and Harris Hawks algorithm. First, we used the variance filter method to select a subset of feature genes. Second, we used the extremely randomized tree to calculate the importance score of each gene and obtain an effective gene subset. Finally, we used Harris Hawks algorithm to obtain the optimal feature gene subset. The pseudocode code of VEH is shown in algorithm 1. Figure 2 shows the gene selection process of the VEH algorithm. In a large dataset, the running time of the wrapper method is usually several orders of magnitude higher than that of the filter method and the embedded method. In the hybrid method VEH, the running time is mainly concentrated on the wrapper method HHO during the third stage, so the time complexity of the proposed method is O(N × (T + T⋅D + 1)).

**Table:** 

**Algorithm 1:** VEH Pseudocode
Inputs: Initial data: *S*(*F*_1_, *F*_2_, ⋯, *F*_*N*_)
Outputs: *X*_*rabbit*_
*S*_1_(*F*_1_, *F*_2_, ⋯, *F*_*N*_)= *VF*(*S*)
E-importance= *ERT*(*S*_1_)
for(int i=0; i<N; i++)
If E-importance[i]>0
add feature i into *hawk*(*X*_*i*_)
endfor
While(stopping condition is not met) do
Calculate the fitness values of hawks
Set *X*_*rabbit*_ as the location of rabbit(best location)
for (each *hawk*(*X*_*i*_)) do
Update the initial energy *E*_0_ and jump strength J
Update the E using Eq.()
if (|*E*| ≥ 1) then //Exploration phase
Update the location vector using Eq.()
if ($ \left\vert E \right\vert \lt 1$) then //Exploitation phase
if(*r* ≥ 0.5 and |*E*| ≥ 0.5) then //soft besiege
Update the location vector using Eq.()
else if(*r* ≥ 0.5 and $ \left\vert E \right\vert \lt 0.5$) then //hard besiege
Update the location vector using Eq.()
else if(*r* < 0.5 and $ \left\vert E \right\vert \geq 0.5$) then //soft besiege with progressive
rapid dives
Update the location vector using Eq.()
else if(*r* < 0.5 and $ \left\vert E \right\vert \lt 0.5$) then //hard besiege with progressive
rapid dives
Update the location vector using Eq.()
Return *X*_*rabbit*_

## Results

### Data collection and experiment setting

In this article, eight microarray gene expression datasets we are used to test the performance of each algorithm. The datasets used were from public websites: http://csse.szu.edu.cn/staff/zhuzx/Datasets.Html (Qu et al., 2021) and https://github.com/Pengeace/MGRFE-GaRFE (Peng et al., 2021). Table 1 provides a detailed overview of the eight microarray datasets, including their sample size, number of genes, and class. In these datasets, the number of genes rangeds from 2,308 to 22,283, and the number of samples was less than 300. These datasets included central nervous system (CNS), leukemia (Leuk), diffuse large B-cell lymphoma (DLBCL), prostate (Pros), gastric2 (Gas2), acute lymphoblastic leukemia 1 (ALL1), ovarian cancer (Ovarian) and small round blue cell tumor (SRBCT). Among these, only SRBCT was a four-class dataset and the others were binary datasets. The number of class samples was uneven except in Prostate and Gas2. Therefore, the datasets used in this article were determined to comprehensively test the performance of different algorithms. During the data preprocessing, in order to operate simply and not change the mean of the variables, we used the mean substitution method to fill in missing values in the dataset and the min-max normalization method to eliminate the impact of data dimensions. All the experimental results in this article were generated on a computer equipped with a corei7-8750 CPU, 16G of memory, and 2.20 GHz frequency. All algorithms were implemented using Python 3.7.0 and two public machine learning libraries, scikit-learn and scikit- feature. In this article, we used three different classifiers, Decision Tree (DT), Support Vector Machine (SVM), and Logistic Regression (LR), to evaluate the performance of each algorithm. The classification performances of each standard classifier were recorded after tenfold cross-validation. The tenfold cross-validation method randomly divide the dataset into 10 parts, nine of which divided into training sets and the rest were divided into test sets. We compared the VEH method with 13 different methods from the literature. The 13 different methods were the *T*-test (T), Wilcoxon-test (Wilcoxon), variance filter-extremely randomized tree (VF-ERT), variance filter-Harris Hawks (VF-HHO), extremely randomized tree-Harris Hawks (ERT-HHO), variance filter-genetic algorithm (VF-GA), extremely randomized tree-genetic algorithm (ERT-GA), variance filter-particle swarm optimization algorithm (VF-PSO), extremely randomized tree-particle swarm optimization algorithm (ERT-PSO). variance filter-crow search algorithm (VF-CSA), extremely randomized tree-crow search algorithm (ERT-CSA), variance filter- differential evolution algorithm (VF-DE), and extremely randomized tree-differential evolution algorithm (ERT-DE), Table 2 lists the specific parameter values of each algorithm and classifier. All experiments were run independently 10 times and used seven evaluation criteria to reflect the performance of each algorithm: the number of selected genes, classification accuracy (Acc), precision rate (precision), recall rate (recall), F1-Score (f1), standard deviation (SD) and algorithm running time. The calculation formulas for the four important evaluation criteria were as follows:

(8) \begin{eqnarray*}\mathrm{Acc}& = \frac{TN+TP}{P+N} \end{eqnarray*}

(9) \begin{eqnarray*}\text{precision}& = \frac{TP}{TP+FP} \end{eqnarray*}

(10) \begin{eqnarray*}\text{recall}& = \frac{TP}{TP+FN} \end{eqnarray*}

(11) \begin{eqnarray*}\mathrm{f}1& =2\ast \frac{\mathit{precision}\ast \mathit{recall}}{\mathit{precision}+\mathit{recall}} \end{eqnarray*}

**Table 1:** Microarray dataset.

No	Dataset	Samples	Genes	Class
1	CNS	60(1:21, 2:39)	7,129	2
2	Leukemia	72(ALL:47, AML:25)	7,129	2
3	DLBCL	77(1:58, 2:19)	7,129	2
4	Prostate	102(1:52, 2:50)	1,2625	2
5	Gastric2	124(1:62, 2:62)	22,283	2
6	ALL1	128(1:95, 2:33)	12,625	2
7	Ovarian	253(Normal:91, Cancer:162)	15,154	2
8	SRBCT	83(1:29, 2:11, 3:18, 4:25)	2,308	4

**Table 2:** Parameters of each algorithm and classifier.

Algorithm	Parameter
VF	Variance Threshold (VT): 0.05
ERT	nEstimators:100, minSamplesLeaf:20, maxLeafNodes:10
T	*α* = 0.0001
Wilcoxon	*α* = 0.0001
HHO	Population size n:30, number of generations T:100
GA	Population size n:30, number of generations T:100, CR: 0.5, MR:0.2
CSA	Population size n:30, number of generations T:100, AP: 0.25, fl:1.5
DE	Population size n:30, number of generations T:100, CR: 0.9
DT	Random state:0, max depth:8, max features:10
SVM	regularization parameter C:1.0, Radial Basis Function
LR	regularization parameter C:1.0, RSP: l2

Number of positive samples (P); Number of negative samples (N). True positive (TP): the real category of the sample is positive, and the model prediction is also positive. True negative (TN): the real category of the sample is a negative case, and the model prediction is also a negative case. False positive (FP): the real category of the sample is negative, and the model prediction is positive. False negative (FN): the real category of the sample is positive and the model prediction is negative. Because the precision, recall, and f1 were for a single class, we assigned the same weight to each class and calculated their average values.

### Comparison with other algorithms

In this section, we comprehensively compared the VEH method with T, VF-ERT, Wilcoxon, VF-HHO, ERT-HHO, VF-GA, ERT-GA, VF-PSO, ERT-PSO, VF-CSA, ERT-CSA, VF-DE and ERT-DE. We performed 10 times tenfold cross-validation on the gene subsets selected by each algorithm to obtain the average result, and the performance optimal values in each dataset are highlighted in black bold. Tables 3–5 shows the performance values of the four evaluation criteria of each algorithm on the three classifiers. Table 3 shows that, on the DT classifier, the VEH method had obvious advantages over other methods, in which the Acc, precision, recall and f1 winning times were 7, 6, 7, and 7, respectively. The VEH average Acc reached 92.42% and achieved 100% classification performance on the Gas2 and ALL1 datasets. As shown in Table 4, on the SVM classifier, the winning times of the VEH method on the four evaluation criteria were 6, 5, 6, and 5, respectively. The VEH achieved 100% classification performance on the Gas2, ALL1, and Ovarian datasets. At the same time, the average Acc reached its best of 95.33%. As shown in Table 5, on the LR classifier, the VEH method has won 7, 4, 5 and 5 times on the four evaluation criteria, respectively. The performance of the four evaluation criteria reached 100% on the Gas2, ALL1 and Ovarian datasets. At the same time, the average Acc was higher than that of the other 13 methods, reaching 95.05%. In summary, compared with the other 13 methods, VEH showed advantages in four evaluation criteria on the three classifiers, especially in the DT classifier, and achieved the highest average Acc in the SVM classifier, which also proves that the hybrid method proposed in this article could deliver an effective and improved performance. Table 6 lists the number of genes selected by each algorithm in the eight datasets. The results show that the VEH method selected the smallest average number of genes. The number of genes selected by the VF-HHO, ERT-HHO, VF-GA, and ERT-GA methods in the five datasets was fewer than that of the VEH method, but as shown in Tables 3–5, the VEH method had significant advantages in many evaluation criteria. In addition, the number of genes selected by the VEH method was only 1/20 to 1/80 of the VF-ERT method, 1/3 to 1/400 of the *T*-test and Wilcoxon-test method, 1/50 to 1/100 of the VF-CSA method, and 1/20 to 1/50 of the ERT-DE method, but it performeds better. The above results show that VEH can better evaluate the correlation between genes through the hybrid method and improve performance. We compared VEH with other algorithms from recent years. Table 7 lists the comparison results between VEH and other methods, and “/” indicates the corresponding missing data. According to the results in Table 7, compared with the existing methods, the VEH method also showed certain competitiveness in Acc.

**Table 3:** Performance comparison of algorithms on classifier DT.

Data-set	Mea-sure	T	VF-ERT	Wil-coxon	VF-HHO	ERT-HHO	VF-GA	ERT-GA	VF-PSO	ERT-PSO	VF-CSA	ERT-CSA	VF-DE	ERT-DE	VEH
CNS	Acc	58.33	64.27	66.67	61.91	63.09	60.71	54.76	66.67	73.33	63.88	58.33	62.50	63.89	**83.36**
	SD	0.00	15.00	0.00	11.64	15.85	12.47	24.93	15.71	19.56	10.09	21.73	8.74	11.39	4.81
	precision	70.83	68.25	74.29	69.22	68.73	74.95	61.63	67.17	76.83	70.56	69.03	71.31	72.39	**84.57**
	recall	58.33	64.28	66.67	61.91	63.09	60.42	54.76	66.67	73.33	63.89	58.33	62.50	63.89	**83.33**
	f1	61.11	65.20	68.75	63.65	64.36	61.84	56.67	66.92	75.04	65.47	60.41	64.43	65.55	83.17
Leuk- emia	Acc	93.33	89.52	86.67	80.95	81.90	86.67	88.57	89.11	91.79	88.89	88.89	90.00	92.22	**95.24**
	SD	0.00	3.56	0.00	11.18	14.25	9.43	5.04	5.80	9.79	5.44	3.44	3.65	5.02	3.25
	precision	**93.94**	91.51	88.89	81.52	68.73	74.95	61.63	91.50	94.08	90.90	90.47	91.68	93.53	84.57
	recall	93.33	89.58	86.67	80.95	81.90	86.67	88.57	88.75	91.25	88.89	88.89	90.00	92.22	**95.23**
	f1	93.12	89.24	85.61	80.13	82.08	86.49	87.80	90.10	92.64	88.75	88.53	89.60	92.00	**95.28**
DL-BCL	Acc	75.00	87.50	93.75	74.57	81.25	82.14	78.57	87.64	89.86	91.67	90.00	90.63	89.58	**94.53**
	SD	0.00	5.10	0.00	12.55	7.22	6.68	11.89	11.04	6.35	3.23	8.39	3.42	5.10	4.01
	precision	83.33	93.99	95.83	86.05	87.58	89.34	88.74	90.68	92.33	93.54	94.21	93.75	93.32	**96.72**
	recall	75.00	87.50	93.75	74.57	81.25	82.14	78.57	87.25	89.25	91.67	90.05	90.63	89.58	**94.64**
	f1	78.21	89.16	94.26	76.98	83.13	83.92	81.70	88.93	90.76	91.79	90.05	91.41	90.63	**95.16**
Pros-tate	Acc	71.43	76.19	74.15	74.15	62.58	72.79	72.79	66.94	71.53	80.95	79.36	79.36	79.37	**81.63**
	SD	0.00	7.77	0.00	8.63	10.80	5.30	6.57	12.61	15.37	6.73	8.87	5.76	7.78	4.28
	precision	71.43	78.38	74.87	74.96	87.57	89.34	88.74	68.75	74.48	81.36	81.09	80.55	81.54	**96.72**
	recall	71.43	76.19	74.15	74.15	62.58	72.79	72.79	66.50	71.50	80.59	79.36	79.36	79.37	**81.63**
	f1	71.43	75.41	74.07	73.91	62.12	72.48	72.32	67.61	72.96	80.86	78.93	78.96	78.93	**81.52**
Gas2	Acc	96.00	96.00	96.00	97.25	95.60	96.42	92.86	92.36	90.27	96.67	96.67	95.33	98.00	**100**
	SD	0.00	2.31	0.00	3.66	4.67	5.48	6.45	5.89	6.68	1.63	3.01	3.27	4.90	0.00
	precision	96.36	96.44	96.36	92.46	91.82	89.91	92.36	93.69	91.12	96.36	97.05	95.64	98.07	**97.28**
	recall	96.00	96.00	96.00	92.00	90.59	89.71	91.43	92.50	90.33	96.00	96.67	95.33	98.00	**97.14**
	f1	96.03	96.03	96.03	92.03	90.62	89.73	91.51	93.09	90.72	96.03	96.67	95.33	98.01	**97.15**
ALL1	Acc	96.15	97.25	96.15	93.96	95.60	94.42	92.86	97.33	98.67	96.80	99.36	96.79	96.16	**100**
	SD	0.00	3.66	0.00	3.02	4.67	5.48	6.45	3.44	2.81	3.78	1.57	2.90	4.21	0.00
	precision	96.70	97.88	96.70	94.68	96.40	96.87	93.16	97.71	98.75	97.53	99.45	97.39	96.48	**100**
	recall	96.15	97.25	96.15	95.60	93.96	96.42	92.86	97.23	98.75	96.80	99.36	96.79	96.16	**100**
	f1	96.25	95.47	96.25	95.47	93.98	96.52	92.88	97.47	98.75	96.93	99.38	96.90	96.21	**100**
Ova-rian	Acc	86.27	**100**	90.20	97.20	92.72	92.30	92.72	96.14	95.75	98.37	94.12	97.39	92.81	98.04
	SD	0.00	0.00	0.00	2.74	5.40	3.17	4.19	5.13	5.57	0.80	3.04	1.01	5.91	1.60
	precision	86.23	**100**	90.41	97.36	93.49	93.42	93.13	96.39	96.12	98.42	94.43	97.47	93.14	98.13
	recall	86.27	**100**	90.20	97.20	92.72	93.00	92.72	96.15	95.77	98.37	94.12	97.39	92.81	98.04
	f1	85.90	**100**	89.93	97.20	92.74	92.82	93.85	96.27	95.94	98.35	94.00	97.37	92.50	98.04
SR-BCT	Acc	81.24	84.88	81.24	65.55	71.43	69.80	68.07	71.25	83.75	70.59	76.47	70.59	76.47	**86.56**
	SD	0.00	4.63	0.00	6.29	7.91	12.72	12.18	13.24	10.29	5.88	8.67	10.57	12.45	4.45
	precision	79.27	87.67	81.97	70.64	73.11	76.50	73.92	62.13	81.25	69.61	81.68	65.88	81.76	**88.49**
	recall	81.24	84.88	81.24	65.55	71.43	71.43	68.07	66.67	82.08	70.59	76.47	70.59	76.47	**86.56**
	f1	79.14	83.91	81.10	64.24	69.18	70.46	67.71	64.32	81.66	69.72	76.59	67.64	74.25	**86.49**
Win-ner	Acc	0	1	0	0	0	0	0	0	0	0	0	0	0	**7**
	precision	1	1	0	0	0	0	0	0	0	0	0	0	0	**6**
	recall	0	1	0	0	0	0	0	0	0	0	0	0	0	**7**
	f1	0	1	0	0	0	0	0	0	0	0	0	0	0	**7**
Mean	Acc	82.22	86.95	85.60	80.69	80.52	81.91	80.15	83.43	86.86	85.98	85.40	85.32	86.06	**92.42**

**Notes.**

The values marked in black and bold represent the best performance values in this dataset.

**Table 4:** Performance comparison of algorithms on classifier SVM.

Data-set	Mea-sure	T	VF-ERT	Wil-coxon	VF-HHO	ERT-HHO	VF-GA	ERT-GA	VF-PSO	ERT-PSO	VF-CSA	ERT-CSA	VF-DE	ERT-DE	VEH
CNS	Acc	66.67	67.86	41.67	53.57	54.76	42.86	53.57	71.67	83.33	62.50	66.67	63.89	62.50	**85.00**
	SD	0.00	5.75	0.00	6.55	15.86	21.21	18.55	19.33	13.61	4.57	7.46	4.31	14.67	14.59
	precision	74.29	70.09	54.17	62.36	68.20	55.22	61.69	67.17	76.83	64.59	73.65	65.28	74.90	**84.92**
	recall	66.67	67.86	41.67	53.57	54.76	42.86	53.64	66.67	73.33	62.50	66.67	63.89	62.50	**78.33**
	f1	68.75	68.31	45.56	55.69	54.72	45.01	55.81	66.92	75.04	63.38	68.42	64.47	64.44	**81.49**
Leuk- emia	Acc	93.33	94.29	86.67	80.95	87.62	91.43	93.33	95.71	86.43	**100**	**100**	**100**	98.89	98.57
	SD	0.00	6.00	0.00	11.82	8.10	6.34	7.70	9.64	11.24	0.00	0.00	0.00	2.72	4.52
	precision	94.13	94.85	90.48	83.15	89.73	92.68	93.59	91.50	90.50	**100**	**100**	**100**	98.99	95.75
	recall	93.33	94.34	86.67	80.95	87.62	91.43	93.33	88.75	85.83	**100**	**100**	**100**	98.89	94.58
	f1	93.24	**96.03**	87.04	80.44	87.50	91.55	93.28	90.10	88.10	**100**	**100**	**100**	98.85	95.16
DL-BCL	Acc	**93.75**	**93.75**	**93.75**	86.36	80.36	81.25	83.04	86.53	89.86	**93.75**	91.67	**93.75**	**93.75**	**93.75**
	SD	0.00	0.00	0.00	7.34	11.08	9.55	12.35	10.19	6.35	0.00	3.23	0.00	0.00	5.10
	precision	95.83	95.83	95.83	92.55	87.77	88.12	92.20	89.32	91.58	95.83	95.14	95.83	95.83	**96.31**
	recall	**93.75**	**93.75**	**93.75**	86.36	80.36	81.25	82.04	86.00	89.25	**93.75**	91.67	**93.75**	**93.75**	**93.75**
	f1	94.26	94.26	94.26	87.97	82.70	83.46	85.57	87.63	90.40	94.26	92.01	94.26	94.26	**94.43**
Pros-tate	Acc	85.71	87.75	85.71	72.11	80.27	80.27	79.59	81.39	85.97	84.92	86.89	84.92	86.66	**87.87**
	SD	0.00	2.55	0.00	7.98	7.49	4.28	4.53	16.42	24.80	1.94	2.46	1.94	2.13	3.60
	precision	85.98	**88.46**	85.98	72.70	81.08	80.77	80.28	68.75	74.48	85.14	86.95	85.14	87.17	87.75
	recall	85.71	87.75	85.71	72.11	80.27	80.27	79.59	66.50	71.50	84.92	85.89	84.92	86.66	**87.87**
	f1	85.65	87.66	85.65	71.92	80.08	80.17	79.48	67.61	72.96	84.87	86.78	84.87	86.59	**87.81**
Gas2	Acc	**100**	**100**	**100**	92.62	93.71	98.86	98.86	97.18	98.09	**100**	**100**	**100**	**100**	**100**
	SD	0.00	0.00	0.00	4.86	4.54	1.95	1.95	4.54	4.03	0.00	0.00	0.00	0.00	0.00
	precision	**100**	**100**	**100**	93.51	94.45	98.96	98.96	93.69	91.12	**100**	**100**	**100**	**100**	**100**
	recall	**100**	**100**	**100**	92.62	93.71	98.86	98.86	92.50	90.33	**100**	**100**	**100**	**100**	**100**
	f1	**100**	**100**	**100**	92.71	93.72	98.87	98.87	93.09	91.23	**100**	**100**	**100**	**100**	**100**
ALL1	Acc	**100**	**100**	**100**	95.05	96.15	97.52	96.70	98.00	98.67	**100**	**100**	**100**	**100**	**100**
	SD	0.00	0.00	0.00	2.91	3.14	2.88	2.65	3.22	2.81	0.00	0.00	0.00	0.00	0.00
	precision	**100**	**100**	**100**	96.89	95.84	97.79	96.91	98.19	98.89	**100**	**100**	**100**	**100**	**100**
	recall	**100**	**100**	**100**	95.05	96.15	97.52	96.70	98.04	98.57	**100**	**100**	**100**	**100**	**100**
	f1	**100**	**100**	**100**	95.19	96.26	97.44	96.70	98.11	98.73	**100**	**100**	**100**	**100**	**100**
Ova-rian	Acc	**100**	**100**	**100**	96.92	98.04	99.19	98.88	99.23	97.66	**100**	**100**	**100**	**100**	**100**
	SD	0.00	0.00	0.00	4.21	1.96	1.01	1.54	1.62	2.01	0.00	0.00	0.00	0.00	0.00
	precision	**100**	**100**	**100**	97.49	98.17	99.20	98.97	96.11	97.83	**100**	**100**	**100**	**100**	**100**
	recall	**100**	**100**	**100**	96.92	98.04	99.16	98.88	95.77	97.66	**100**	**100**	**100**	**100**	**100**
	f1	**100**	**100**	**100**	96.98	98.04	99.16	98.89	95.94	97.74	**100**	**100**	**100**	**100**	**100**
SR-BCT	Acc	76.47	**100**	85.56	84.04	89.08	75.63	86.56	90.00	83.75	82.35	88.24	83.82	82.35	97.48
	SD	0.00	0.00	0.00	11.62	6.29	17.79	13.46	7.91	10.29	6.47	9.31	5.63	6.71	4.62
	precision	86.93	**100**	87.08	82.64	90.53	78.34	88.26	62.13	86.25	87.25	90.39	79.50	85.71	98.23
	recall	76.47	**100**	86.56	84.04	89.08	75.63	86.56	66.67	85.42	82.35	88.24	83.82	88.24	97.48
	f1	75.13	**100**	86.12	82.48	88.94	74.69	86.66	64.32	85.83	81.39	87.85	80.99	85.85	97.59
Win-ner	Acc	4	5	4	0	0	0	0	0	0	5	4	5	4	**6**
	precision	3	5	3	0	0	0	0	0	0	4	4	4	3	**5**
	recall	4	4	4	0	0	0	0	0	0	5	4	5	4	**6**
	f1	3	5	3	0	0	0	0	0	0	4	4	4	3	**5**
Mean	Acc	89.49	92.96	86.67	82.70	85.00	83.38	86.32	89.96	90.47	90.44	91.68	90.80	90.52	**95.33**

**Notes.**

The values marked in black and bold represent the best performance values in this dataset.

**Table 5:** Performance comparison of algorithms on classifier LR.

Data-set	Mea-sure	T	VF-ERT	Wil-coxon	VF-HHO	ERT-HHO	VF-GA	ERT-GA	VF-PSO	ERT-PSO	VF-CSA	ERT-CSA	VF-DE	ERT-DE	VEH
CNS	Acc	66.67	65.48	50.00	52.38	57.14	39.29	59.52	68.33	75.00	69.45	70.83	69.44	68.06	**76.67**
	SD	0.00	5.75	0.00	15.75	13.97	12.47	18.28	18.34	14.16	8.61	10.21	12.55	6.27	17.92
	precision	**85.71**	65.69	58.57	60.48	72.22	55.22	71.55	80.00	83.83	69.72	76.28	73.44	71.81	75.83
	recall	66.67	65.48	50.00	52.38	57.14	39.29	59.52	78.33	80.00	69.45	70.83	71.11	68.06	**76.67**
	f1	68.57	65.44	53.13	51.80	58.72	42.65	61.91	79.16	81.87	69.43	72.29	70.66	68.49	**76.25**
Leuk- emia	Acc	86.67	94.29	94.29	91.43	83.81	88.57	91.43	94.46	93.21	**100**	**100**	**100**	**100**	98.09
	SD	0.00	6.00	0.00	7.42	8.48	10.69	8.36	7.17	7.18	0.00	0.00	0.00	0.00	3.25
	precision	90.48	95.16	94.60	92.50	86.68	89.20	92.14	95.75	94.75	**100**	**100**	**100**	**100**	98.41
	recall	86.67	94.29	94.29	91.43	83.81	88.57	91.40	94.17	92.92	**100**	**100**	**100**	**100**	98.09
	f1	87.04	93.97	94.32	91.57	84.09	88.64	91.77	94.95	93.83	**100**	**100**	**100**	**100**	98.13
DL-BCL	Acc	87.50	**92.85**	87.50	74.82	78.57	79.46	78.57	88.61	88.75	91.75	89.58	91.75	89.58	**92.85**
	SD	0.00	2.36	0.00	8.70	11.33	10.65	8.73	7.43	9.08	3.21	3.23	4.31	3.23	2.36
	precision	93.75	**95.53**	93.75	88.27	90.38	91.52	90.95	90.65	91.15	92.83	92.44	92.83	92.44	93.47
	recall	87.50	**92.86**	87.50	74.82	78.57	79.46	78.57	88.25	88.25	91.75	89.58	91.75	89.58	91.96
	f1	89.10	**93.52**	89.10	78.02	81.45	82.52	81.94	89.43	89.68	92.26	90.82	92.26	90.82	92.58
Pros-tate	Acc	85.71	87.75	85.71	68.03	78.91	82.99	74.15	90.69	90.56	88.89	89.68	85.71	86.66	**94.03**
	SD	0.00	2.55	0.00	5.97	9.06	4.65	7.70	9.31	9.35	2.46	3.59	4.26	2.13	8.64
	precision	85.98	88.53	85.98	68.38	79.85	83.92	74.61	68.75	74.48	89.95	90.57	86.29	87.17	**90.89**
	recall	85.71	87.75	85.71	68.03	78.91	82.99	74.15	66.50	71.50	88.89	89.68	85.71	86.66	**89.80**
	f1	85.65	87.66	85.65	67.99	78.71	82.76	74.13	67.61	72.96	88.78	89.59	85.65	86.59	**89.65**
Gas2	Acc	**100**	**100**	**100**	95.45	90.86	95.47	97.71	96.18	97.09	**100**	**100**	**100**	**100**	**100**
	SD	0.00	0.00	0.00	5.86	9.44	4.24	2.14	4.94	4.69	0	0	0	0	0.00
	precision	**100**	**100**	**100**	95.73	91.83	96.32	97.92	93.69	91.12	**100**	**100**	**100**	**100**	**100**
	recall	**100**	**100**	**100**	95.45	90.86	95.47	97.71	92.50	90.33	**100**	**100**	**100**	**100**	**100**
	f1	**100**	**100**	**100**	95.49	90.92	95.47	97.73	93.09	90.72	**100**	**100**	**100**	**100**	**100**
ALL1	Acc	**100**	**100**	**100**	92.86	94.50	98.90	94.51	98.67	99.33	**100**	**100**	**100**	**100**	**100**
	SD	0.00	0.00	0.00	6.44	4.89	2.91	5.37	2.81	2.11	0	0	0	0	0.00
	precision	**100**	**100**	**100**	95.07	95.97	99.18	95.88	98.75	99.44	**100**	**100**	**100**	**100**	**100**
	recall	**100**	**100**	**100**	92.86	94.50	98.90	94.51	98.75	99.29	**100**	**100**	**100**	**100**	**100**
	f1	**100**	**100**	**100**	93.14	94.76	98.95	94.74	98.75	99.36	**100**	**100**	**100**	**100**	**100**
Ova-rian	Acc	**100**	**100**	**100**	99.16	99.16	98.04	**100**	99.62	97.26	**100**	**100**	**100**	**100**	**100**
	SD	0.00	0.00	0.00	1.05	1.54	1.96	0.00	1.22	2.62	0	0	0	0	0.00
	precision	**100**	**100**	**100**	99.21	99.23	98.13	**100**	96.12	97.39	**100**	**100**	**100**	**100**	**100**
	recall	**100**	**100**	**100**	99.16	99.16	98.04	**100**	95.77	97.28	**100**	**100**	**100**	**100**	**100**
	f1	**100**	**100**	**100**	99.16	99.17	98.05	**100**	95.94	97.33	**100**	**100**	**100**	**100**	**100**
SR-BCT	Acc	76.47	98.32	78.15	76.47	84.03	73.95	84.04	93.75	90.00	88.24	84.12	82.16	88.24	**98.75**
	SD	0.00	2.87	0.00	11.77	15.82	10.66	12.58	8.84	11.49	7.35	8.67	3.40	5.89	3.95
	precision	86.93	**98.60**	81.82	79.94	92.33	81.23	87.07	62.13	85.83	89.41	79.22	82.35	87.45	94.17
	recall	76.47	**98.32**	78.15	76.47	84.03	73.95	84.04	66.67	88.75	88.24	84.12	81.18	88.24	95.42
	f1	75.13	**98.33**	77.12	76.42	85.52	75.03	83.94	64.32	87.27	88.34	81.44	81.23	87.62	94.79
Win-ner	Acc	3	4	3	0	0	0	1	0	0	4	4	4	4	**7**
	precision	4	5	3	0	0	0	1	0	0	4	4	4	4	**4**
	recall	3	5	3	0	0	0	1	0	0	4	4	4	4	**5**
	f1	3	5	3	0	0	0	1	0	0	4	4	4	4	**5**
Mean	Acc	87.88	92.34	86.96	81.32	83.37	82.08	84.99	91.29	91.40	92.29	91.78	91.13	91.57	**95.05**

**Notes.**

The values marked in black and bold represent the best performance values in this dataset.

**Table 6:** Comparison between the VEH based on the number of selected genes and other methods.

Data -set	Mea-sure	T	VF- ERT	Wilcoxon	VF- HHO	ERT-HHO	VF-GA	ERT-GA	VF- PSO	ERT-PSO	VF- CSA	ERT- CSA	VF-DE	ERT-DE	VEH
CNS	Mean	16	424.42	**3**	6.14	12.71	6.86	6.71	375.67	188.3	392.0	226.8	360.2	183.0	5.14
	SD	0	100.77	0	6.09	7.20	1.57	1.25	18.39	11.60	8.67	5.27	21.58	17.1	3.85
Leuk-emia	Mean	511	335.71	402	**3.71**	**3.71**	6	5.29	324.67	104.0	1425	127.2	1272	96.6	6.29
	SD	0	6.55	0	3.15	1.89	1.63	1.25	27.52	13.43	42.38	11.97	33.54	9.02	2.63
DLBCL	Mean	896	221.57	889	14.43	7.14	5.57	6.71	441.17	122.2	549.8	137.8	515.6	98.86	**5.14**
	SD	0	14.58	0	12.71	7.24	1.27	1.89	39.49	8.98	18.64	9.33	29.10	5.46	2.61
Pros- tate	Mean	100	513.14	89	**4.86**	9.57	6.86	6.43	682.17	265.7	622.5	287.6	554.8	261.5	6.71
	SD	0	14.11	0	2.61	8.24	1.57	1.13	18.37	16.67	22.23	10.26	21.6	14.77	2.29
Gas2	Mean	8066	296.57	8058	3.71	**3.29**	6.29	7.57	791.00	129.3	878.7	150.2	786.3	116.1	5
	SD	0	9.46	0	3.73	1.70	2.36	2.30	44.42	15.42	10.52	10.38	7.74	10.23	2.31
ALL1	Mean	1972	100	1915	**1.71**	2.86	4.57	5.86	244.83	118.0	350.0	135.0	295.2	90.17	2.86
	SD	0	2.65	0	0.95	3.63	2.57	2.73	22.50	11.03	24.75	8.20	7.25	8.47	1.22
Ova-rian	Mean	5235	99.14	5169	16.86	9.29	**5.29**	7.29	213.67	339.3	180.7	343.5	153.0	294.3	5.57
	SD	0	5.46	0	18.35	8.24	2.22	0.95	14.22	26.49	9.77	14.17	4.20	11.88	2.23
SRB-CT	Mean	55	140.29	65	23.57	22.57	8.86	8.57	157.50	213.5	179.0	218.6	168.8	179.3	**7.57**
	SD	0	6.40	0	11.63	9.13	1.22	1.27	13.81	24.79	10.86	9.37	8.66	8.09	2.44
Mean		2106	266.36	2074	9.38	8.89	6.29	6.80	403.84	185.1	572.2	203.3	513.2	165.0	**5.54**

**Notes.**

The values marked in black and bold represent the best performance values in this dataset.

**Table 7:** Comparison between the proposed method and other methods in Acc.

Methods	CNS	Leukemia	DLBCL	Prostate	Gas2	ALL1	Ovarian	SRBCT
MGRFE (Peng et al., 2021)	/	91.10	/	78.30	95.60	100	/	/
BIRS (Wang, Wang & Chang, 2016)	64.14	/	/	/	/	/	98.50	86.66
BCROSAT (Salcedo-Sanz et al., 2014)	81.67	/	/	/	/	/	/	95.76
CFS (Deng et al., 2022)	68.33	76.25	67.50	89.27	/	/	/	/
IGA-FBFE (Aziz, Verma & Srivastava, 2016)	/	94.20	/	88.12	/	/	/	/
IWSSr (Attiya, Abd Elaziz & Xiong, 2020)	/	87.50	81.23	78.70	/	/	/	/
FCSVM-RFE (Deng et al., 2022)	58.33	79.11	/	78.27	/	/	95.29	/
Grasp-IWSSr (Pirgazi et al., 2019)	/	91.60	85.61	77.50	/	/	/	/
Pso-Dica (Nguyen et al., 2020)	/	88.89	/	/	/	/	/	96.00
Propose	**85.00**	**98.57**	**94.53**	**94.03**	**100**	**100**	**100**	**98.75**

**Notes.**

The values marked in black and bold represent the best performance values in this dataset.

Table 8 lists the average running time of each algorithm on each dataset. By comparing the results, we found that the Wilcoxon-test method had the shortest running time and the VF-PSO method had the longest running time. In combination with the results in Tables 3–7, we found that although the VF-ERT, *T*-test, and Wilcoxon-test methods had a short run time, this was at the expense of Acc and the number of selected genes. The VF-HHO, ERT-HHO, VF-GA, ERT-GA, VF-PSO, ERT-PSO, VF-CSA, ERT-CSA, VF-DE and ERT-DE methods had a long run time, but their other performance was significantly lower than VEH. This also shows that VEH can effectively improve performance and shorten the overall run time by combining various methods. Of all the evaluation criteria, Acc was the most important, so we tested the performance of the VEH method in the dataset when *α* tooks different values. As shown in Table 9, when *α* = 0.99, the algorithm performance was the best. Therefore, we set *α* = 0.99.

**Table 8:** Comparison of the running time (100*S*) between the VEH and other methods.

Dataset	T	VF-ERT	Wil-coxon	VF-HHO	ERT-HHO	VF-GA	ERT-GA	VF-PSO	ERT-PSO	VF-CSA	ERT-CSA	VF-DE	ERT-DE	VEH
CNS	17.22	**10.65**	17.27	54.15	36.84	33.06	54.19	51.83	42.65	49.49	36.88	36.00	26.57	25.63
Leuk	18.72	**9.86**	17.71	116.12	35.19	30.91	62.31	149.61	33.66	130.2	36.53	86.65	21.38	29.17
DLBCL	21.44	**11.12**	19.95	60.52	8.99	34.30	60.44	73.56	48.45	39.11	21.34	44.19	22.70	27.97
Prostate	10.03	**6.56**	9.15	64.97	36.30	34.51	35.73	85.73	51.61	78.62	52.06	47.60	32.51	24.10
Gas2	99.10	**61.22**	83.55	128.22	86.72	83.35	180.14	179.30	118.86	68.42	28.77	111.2	67.48	76.31
ALL1	38.30	70.67	**34.53**	105.44	96.67	89.23	180.22	128.95	118.86	172.9	153.1	136.7	129.1	87.42
Ovarian	90.82	148.05	**84.44**	188.49	198.57	178.27	282.35	200.29	219.13	170.53	189.1	139.1	152.7	174.22
SRBCT	6.59	**5.96**	7.15	33.06	42.05	28.66	29.98	36.44	37.50	39.46	44.74	23.38	27.65	24.07
Mean	37.78	40.51	**34.22**	93.87	67.67	64.04	110.67	113.21	83.84	93.59	70.32	78.12	60.01	58.61

**Notes.**

The values marked in black and bold represent the best performance values in this dataset.

**Table 9:** Comparison of Acc between different *α* values in VEH.

Dataset	Measure	0.1	0.2	0.3	0.4	0.5	0.6	0.7	0.8	0.9	0.99
CNS	DT	64.58	52.78	64.58	54.17	55.56	66.67	70.84	72.22	77.78	**83.36**
	SVM	64.58	66.67	58.33	56.25	63.89	63.89	63.89	66.67	79.16	**85.00**
	LR	50.00	50.00	44.44	62.50	66.67	63.89	66.66	70.84	75	**76.67**
Leuk	DT	85.00	80.00	78.36	81.67	86.67	85.00	86.67	88.89	90.00	**95.24**
	SVM	80.00	75.56	80.00	80.00	85.00	86.67	86.67	93.33	93.33	**98.57**
	LR	80.00	80.00	81.67	81.67	88.89	88.89	88.89	90.00	93.33	**98.09**
DLBCL	DT	82.81	85.42	81.25	85.94	77.00	85.94	90.63	90.63	93.75	**94.53**
	SVM	81.25	85.42	83.33	82.81	81.25	85.94	85.94	87.50	90.63	**93.75**
	LR	77.08	84.38	84.38	81.25	81.25	84.38	87.50	87.50	90.63	**92.85**
Prostate	DT	74.60	69.84	69.05	66.67	73.02	76.19	77.38	77.78	80.95	**81.63**
	SVM	73.02	77.38	77.38	74.60	77.38	80.95	79.36	82.14	85.71	**87.87**
	LR	75.00	77.38	74.60	77.78	76.19	79.76	80.95	85.71	90.48	**94.03**
Gas2	DT	90.00	85.00	95.00	95.00	91.00	93.00	97.00	98.00	**100**	**100**
	SVM	93.00	92.00	96.00	94.00	93.00	95.00	97.00	96.00	**100**	**100**
	LR	93.00	96.00	94.00	92.00	94.00	97.00	97.00	99.00	99.00	**100**
ALL1	DT	92.31	95.19	92.31	96.15	98.08	95.19	94.23	95.19	**100**	**100**
	SVM	92.31	97.12	94.23	96.15	92.31	96.15	94.23	98.08	**100**	**100**
	LR	91.35	94.23	95.19	93.27	93.27	93.27	98.08	98.08	**100**	**100**
Ovarian	DT	94.12	96.08	95.10	94.12	94.12	94.12	94.12	97.55	97.00	**98.04**
	SVM	96.57	97.06	96.08	95.10	**100**	96.08	99.02	**100**	**100**	**100**
	LR	96.08	94.12	99.02	99.51	93.63	99.51	99.51	98.04	**100**	**100**
SRBCT	DT	64.71	64.71	76.48	70.59	70.59	76.47	82.35	76.47	82.35	**86.56**
	SVM	70.59	76.47	88.24	82.35	86.27	94.12	88.24	88.24	94.12	**97.48**
	LR	70.59	76.47	82.35	84.31	82.35	76.47	94.12	88.24	94.12	**98.75**
Winner	DT	0	0	0	0	0	0	0	0	2	8
	SVM	0	0	0	0	1	0	0	1	3	8
	LR	0	0	0	0	0	0	0	0	2	8

**Notes.**

The values marked in black and bold represent the best performance values in this dataset.

### Gene analysis

Because the VEH method had certain randomness, we may have seen the same performance during the process of feature selection. To address this issue, we followed the following principles: (1) select the high Acc subset; (2) when the Acc is the same, select small subsets; and (3) when the Acc and the number of subsets are the same, select the highest frequency. Table 10 lists the optimal number of gene subsets, probe/Uniprot ID, and Acc on different classifiers in each dataset. We have biologically described the best subset of genes selected in five datasets, and the corresponding results are listed in Tables 11–15. Liddelow & Hoyer (2016) found that NCAM1 represents a potential drug target for many inflammatory diseases of the CNS. Clark & Stein (2020) also found that CD33 can target leukemia. Tanhaei et al. (2014) found that GAPDH can be used as a valuable indicator to distinguish DLBCL. Li, Ge & Franceschi (2021) found that RUNX2 plays a key role in the development of the prostate. Hu et al. (2016) found that the EPOR pathway can promote the formation, proliferation, and migration of Gas2.

**Table 10:** Optimal subset of genes selected by the proposed method.

Dataset	Number	Probe/uniprot ID	DT	SVM	LR
CNS	4	M22092_at,M33521_at,U28687_at U95740_rna1_at	91.67	83.33	66.67
Leukemia	4	J05243_at,M23197_at, U79296_at U05259_rna1_at	93.33	100	100
DLBCL	8	AFFX-HUMGAPDH/M33197_5_st L42324_at,L49209_s_at,U00957_at X60955_s_at,X67951_at U19495_s_at,U89922_s_at	93.75	100	93.75
Prostate	7	1060_g_at,1315_at,198_g_at, 31509_at,32210_at,32242_at, 33102_at	85.71	90.48	100
Gastric2	5	396_f_at,202726_at,207392_x_at 212353_at,212462_at	100	100	100
ALL1	2	33039_at,41609_at	100	100	100
Ovarian	8	MZ0.008796743,MZ28.202695 MZ244.95245,MZ290.41236 MZ554.4233,MZ674.57738 MZ4101.0731,MZ8607.049	100	100	100
SRBCT	10	gene3,gene74,gene246 ,gene749 gene836 ,gene1084,gene1093 gene1210,gene1389,gene2186	88.24	100	100

**Table 11:** Description of CNS genes selected by the proposed method.

Probe/uniprot ID	Gene	Description
M22092_at	NCAM1	neural cell adhesion molecule 1
M33521_at	BAG6	BAG6 cochaperone 6
U28687_at	ZNF157	zinc finger protein 157
U95740_rna1_at	MARF1	meiosis regulator and mRNA stability factor 1

**Table 12:** Description of leukemia genes selected by the proposed method.

Probe/uniprot ID	Gene	Description
J05243_at	SPTAN1	spectrin alpha, non-erythrocytic 1
M23197_at	CD33	CD33 molecule
U79296_at	PDHX	pyruvate dehydrogenase complex component X
U05259_rna1_at	CD79A	CD79a molecule

**Table 13:** Description of DLBCL genes selected by the proposed method.

Probe/uniprot ID	Gene	Description
M33197_5_st	GAPDH	glyceraldehyde-3-phosphate dehydrogenase
L42324_at	GPR18	Gprotein-coupled receptor 18
L49209_s_at	pRb	RB transcriptional corepressor 1
U00957_at	PRKA10	A-kinase anchoring protein 10
X60955_s_at	TYRP1	tyrosinase related protein 1
X67951_at	PRDX1	peroxiredoxin 1
U19495_s_at	CXCL12	*C* − *X* − *C* motif chemokine ligand 12
U89922_s_at	LTB	lymphotoxin beta

**Table 14:** Description of prostate genes selected by the proposed method.

Probe/uniprot ID	Gene	Description
1060_g_at	CENPC	centromere protein C
1315_at	COPB	COPI coat complex subunit beta 1
198_g_at	RUNX2	RUNX family transcription factor 2
31509_at	CG12239	Is expressed in embryonic brain
32210_at	COX1	cytochrome c oxidase subunit I
32242_at	mRpL49	mitochondrial ribosomal protein L49
33102_at	CG1494	Predicted to be involved in lipid transport

**Table 15:** Description of Gastric2 genes selected by the proposed method.

Probe/uniprot ID	Gene	Description
396_f_at	EPOR	erythropoietin receptor
202726_at	LIG1	DNA ligase 1
207392_x_at	UGT2B15	UDP glucuronosyltransferase family 2 member B15
212353_at	SULF1	sulfatase 1
212462_at	KAT6B	lysine acetyltransferase 6B

## Discussion

The purpose of VEH is to select effective feature genes from high-dimensional gene expression data. Unlike other similar methods (Bir-Jmel, Douiri & Elbernoussi, 2019; Ge et al., 2016), VEH is a three-stage hybrid method that combines three different methods. The results in Tables 11–15 show that our method can select important genes related to a tumor in multiple datasets (Endo et al., 2018; Forgione et al., 2020), and the results of other studies also verify the effectiveness and practicability of genes selected using the VEH method from a medical perspective.

The results in Tables 3–8 show that VEH significantly improves performance while reducing run time. As shown in Table 9, we also tested the performance value of the VEH method on different datasets when *α* takes different values, which proved the rationality of our *α* value. By combining the filter method and wrapper method, VEH selects key genes after quickly screening redundant genes in a large range, which also shows that our method can improve performance and run time. Simple operation and flexible combination are also important advantages of our method.

## Conclusion

VEH combines the advantages of the filter and wrapper methods. In a variety of tumor gene expression datasets, the average Acc of VEH reached 95.33%. Compared with other algorithms, this method had obvious advantages in Acc, precision, recall, f1, and run time. In future research, we will consider the progress of the Harris Hawk algorithm, how to improve its performance in gene selection, and increase its testing across different datasets.

##  Supplemental Information

10.7717/peerj-cs.1229/supp-1 Supplemental Information 1 Dataset 1: ALL1, CNS, DLBCL and Gastric2

10.7717/peerj-cs.1229/supp-2 Supplemental Information 2 Dataset 2: Leukemia, Ovarian, Prostate and SRBCT

10.7717/peerj-cs.1229/supp-3 Supplemental Information 3 Main codes of each algorithm

## Additional Information and Declarations

### Data Availability

The following information was supplied regarding data availability: The data and code are available in the Supplemental Files.
